# Cs_3_ScCl_6_

**DOI:** 10.1107/S1600536814009799

**Published:** 2014-05-10

**Authors:** Matthew D. Ward, James A. Ibers

**Affiliations:** a Department of Chemistry, Northwestern University, 2145 Sheridan Rd, Evanston, IL 60208-3113, USA

## Abstract

Crystals of tricaesium scandium(III) hexa­chloride were obtained as a side product from the reaction of U, SnCl_2_, Sc, and S in a CsCl flux at 1073 K. Cs_3_ScCl_6_ crystallizes in the Rb_3_YCl_6_ structure type. The asymmetric unit comprises three Cs sites, two Sc sites, and six Cl sites, all of which have site symmetry 1, except for the two Sc sites that have site symmetries of 2 and -1, respectively. The structure is composed of isolated [ScCl_6_]^3−^ octa­hedra that are surrounded by Cs^+^ cations. Two Cs^+^ cations have inter­actions with eight Cl^−^ anions, while the third has inter­actions with ten Cl^−^ anions.

## Related literature   

Cs_3_ScCl_6_ crystallizes in the Rb_3_YCl_6_ structure type (space group *C*2*/c*; Mattfeld & Meyer, 1992 ▶). Previously, a number of ternary scandium halides of the composition *A*_3_Sc*X*_6_ were characterized by single-crystal X-ray diffraction or Rietveld refinements from powder data. These include Na_3_ScF_6_ (Dahlke & Babel, 1994 ▶), Na_3_ScBr_6_ (Bohnsack & Meyer, 1996 ▶), Li_3_ScCl_6_ (Bohnsack *et al.*, 1997 ▶), Li_3_ScF_6_ (Tyagi *et al.*, 2005 ▶), K_3_ScCl_6_ (Cerny *et al.*, 2010*a* ▶), and Na_3_ScCl_6_ (Cerny *et al.*, 2010*b* ▶). Except for Li_3_ScCl_6_, these compounds crystallize in one of two structure types: Na_3_CrCl_6_ (*P* 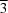
1*c*; Friedrich *et al.*, 1987 ▶) or Na_3_AlF_6_ (*P*2_1_ */n*; Náray-Szabó & Sasvári, 1938 ▶). For other caesium scandium chloride compounds, see: Poeppelmeier *et al.* (1980 ▶). For standardization of structure data, see: Gelato & Parthé (1987 ▶).

## Experimental   

### 

#### Crystal data  

Cs_3_ScCl_6_
*M* *_r_* = 656.39
Monoclinic, 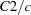

*a* = 26.3310 (5) Å
*b* = 7.9632 (2) Å
*c* = 12.7085 (3) Å
β = 100.006 (1)°
*V* = 2624.17 (10) Å^3^
*Z* = 8
Mo *K*α radiation
μ = 9.93 mm^−1^
*T* = 100 K
0.05 × 0.04 × 0.03 mm

#### Data collection  

Bruker APEXII CCD diffractometer
Absorption correction: numerical (*SADABS*; Bruker, 2009 ▶) *T*_min_ = 0.341, *T*_max_ = 0.438
30195 measured reflections
6379 independent reflections
5631 reflections with *I* > 2σ(*I*)
*R*_int_ = 0.038

#### Refinement  

*R*[*F* ^2^ > 2σ(*F* ^2^)] = 0.023
*wR*(*F* ^2^) = 0.050
*S* = 1.33
6379 reflections
93 parameters
Δρ_max_ = 0.98 e Å^−3^
Δρ_min_ = −1.26 e Å^−3^

### 

Data collection: *APEX2* (Bruker, 2009 ▶); cell refinement: *SAINT-Plus* (Bruker, 2009 ▶); data reduction: *SAINT-Plus*; program(s) used to solve structure: *SHELXS2014* (Sheldrick, 2008 ▶); program(s) used to refine structure: *SHELXL2014*; molecular graphics: *CrystalMaker* (Palmer, 2013 ▶); software used to prepare material for publication: *SHELXL2014*.

## Supplementary Material

Crystal structure: contains datablock(s) I, New_Global_Publ_Block. DOI: 10.1107/S1600536814009799/wm5022sup1.cif

Structure factors: contains datablock(s) I. DOI: 10.1107/S1600536814009799/wm5022Isup2.hkl

CCDC reference: 1000441

Additional supporting information:  crystallographic information; 3D view; checkCIF report

## Figures and Tables

**Fig. 1.**
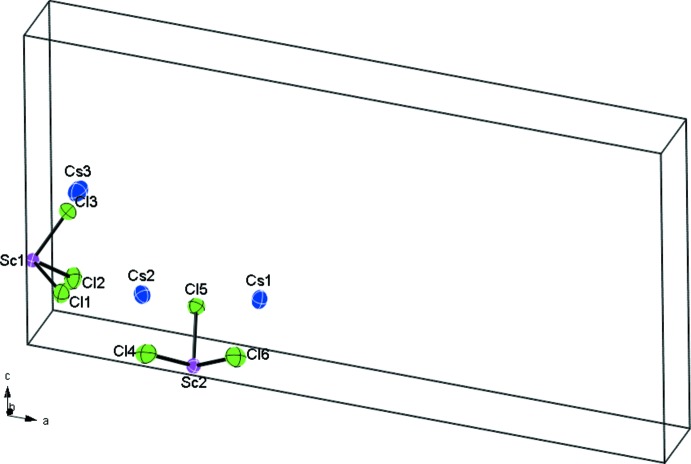
The asymmetric unit of Cs3ScCl6. Displacement ellipsoids at the 99% probability level are shown. Caesium atoms are blue, scandium atoms are purple, and chlorine atoms are green.

**Fig. 2.**
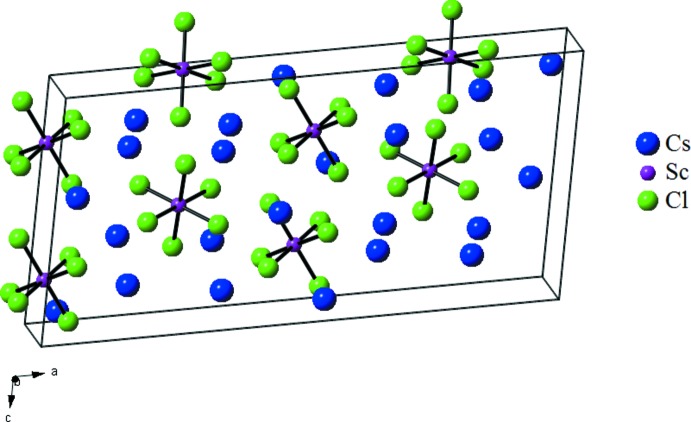
Packing of Cs3ScCl6 viewed nearly along the b axis.

## supplementary crystallographic information

### 1. Comment

Ternary scandium halides of the composition *A*_3_Sc*X*_6_ are known for *A* = Li, Na, K and *X* = F, Cl, Br. These compounds crystallize in either the Na_3_CrCl_6_ (*P*31*c*) (Friedrich *et al.*, 1987) or the Na_3_AlF_6_ (*P*2_1_*/n*) (Náray-Szabó & Sasvári, 1938) structure types, except for Li_3_ScCl_6_ which crystallizes in space group *C*2*/m* (Bohnsack *et al.*, 1997). Single-crystal refinements have been carried out for Na_3_ScF_6_ (Dahlke & Babel, 1994), Na_3_ScBr_6_ (Bohnsack & Meyer, 1996), Li_3_ScCl_6_ (Bohnsack *et al.*, 1997), and Li_3_ScF_6_ (Tyagi *et al.*, 2005). The structures of K_3_ScCl_6_ (Cerny *et al.*, 2010*a*) and Na_3_ScCl_6_ (Cerny *et al.*, 2010*b*) were determined by Rietveld refinement of X-ray powder data. Cs_3_ScCl_6_ is the first Cs-containing compound of the *A*_3_Sc*X*_6_ family. It crystallizes in the monoclinic space group *C*2*/c* in the Rb_3_YCl_6_ structure type (Mattfeld & Meyer, 1992).

The structure of Cs_3_ScCl_6_ is composed of isolated [ScCl_6_]^3-^ octahedra that are surrounded by Cs^+^ cations. The asymmetric unit, comprising three Cs, two Sc, and six Cl sites, is shown in Fig. 1, and a packing diagram is shown in Fig. 2. The composition achieves charge balance by assigning formal oxidation states of +1, +3, and -1 to Cs, Sc, and Cl, respectively. The Sc—Cl distances range from 2.4718 (5) Å to 2.5072 (6) Å at 100 K. These distances compare favorably with those of 2.601 Å at 298 K in Cs_3_Sc_2_Cl_9_ (Poeppelmeier *et al.*, 1980). Another caesium scandium chloride compound is CsScCl_3_ (Poeppelmeier *et al.*, 1980), which reportedly contains Sc(II).

### 2. Experimental

A reaction mixture containing U (0.126 mmol), SnCl_2_ (0.126 mmol), Sc (1.290 mmol), S (0.378 mmol), and CsCl (0.594 mmol) was loaded into a carbon-coated fused-silica tube under an inert Ar atmosphere. The tube was then evacuated to 10 ^-4^ Torr, flame sealed, and placed in a computer-controlled furnace. The tube was heated to 1073 K in 12 h, held there for 96 h, cooled to 773 K at a rate of 2 K/h, and then cooled to 298 K over a further 48 h. The reaction yielded black rectangular prisms of ScU_8_S_17_, purple blocks of Cs_3_ScCl_6_, and excess CsCl flux. Crystals of Cs_3_ScCl_6_ were also found as side products in other reactions that contained Sc and a CsCl flux. Crystals of Cs_3_ScCl_6_ are soluble in water.

### 3. Refinement

Atomic positions were standardized with the program *STRUCTURE TIDY* (Gelato & Parthé, 1987). The highest peak of 1.0 (2) e^-^/Å^3^ is 2.18 Å from atom Cs2 and the deepest hole of -1.3 (2) e^-^/Å^3^ is 0.92 Å from atom Cs3.

### Crystal data

**Table:**

Cs_3_ScCl_6_	*F*(000) = 2304
*M**_r_* = 656.39	*D*_x_ = 3.323 Mg m^−^^3^
Monoclinic, *C*2/*c*	Mo *K*α radiation, λ = 0.71073 Å
*a* = 26.3310 (5) Å	Cell parameters from 9931 reflections
*b* = 7.9632 (2) Å	θ = 3.1–36.3°
*c* = 12.7085 (3) Å	µ = 9.93 mm^−^^1^
β = 100.006 (1)°	*T* = 100 K
*V* = 2624.17 (10) Å^3^	Block, purple
*Z* = 8	0.05 × 0.04 × 0.03 mm

### Data collection

**Table:**

Bruker APEXII CCD diffractometer	5631 reflections with *I* > 2σ(*I*)
φ and ω scans	*R*_int_ = 0.038
Absorption correction: numerical (*SADABS*; Bruker, 2009)	θ_max_ = 36.4°, θ_min_ = 1.6°
*T*_min_ = 0.341, *T*_max_ = 0.438	*h* = −39→43
30195 measured reflections	*k* = −13→12
6379 independent reflections	*l* = −20→21

### Refinement

**Table:**

Refinement on *F*^2^	93 parameters
Least-squares matrix: full	0 restraints
*R*[*F*^2^ > 2σ(*F*^2^)] = 0.023	*w* = 1/[σ^2^(*F*_o_^2^) + (0.0114*F*_o_^2^)^2^]
*wR*(*F*^2^) = 0.050	(Δ/σ)_max_ = 0.002
*S* = 1.33	Δρ_max_ = 0.98 e Å^−^^3^
6379 reflections	Δρ_min_ = −1.26 e Å^−^^3^

### Special details

**Table:**

Geometry. All e.s.d.'s (except the e.s.d. in the dihedral angle between two l.s. planes) are estimated using the full covariance matrix. The cell e.s.d.'s are taken into account individually in the estimation of e.s.d.'s in distances, angles and torsion angles; correlations between e.s.d.'s in cell parameters are only used when they are defined by crystal symmetry. An approximate (isotropic) treatment of cell e.s.d.'s is used for estimating e.s.d.'s involving l.s. planes.

### Fractional atomic coordinates and isotropic or equivalent isotropic displacement parameters (Å^2^)

**Table:**

	*x*	*y*	*z*	*U*_iso_*/*U*_eq_	
Cs1	0.16275 (2)	0.18961 (2)	0.30059 (2)	0.01108 (3)	
Cs2	0.34717 (2)	0.18469 (2)	0.35501 (2)	0.01108 (3)	
Cs3	0.44859 (2)	0.24984 (2)	0.06656 (2)	0.01686 (3)	
Sc1	0.0000	0.22061 (6)	0.2500	0.00772 (8)	
Sc2	0.2500	0.2500	0.0000	0.00814 (8)	
Cl1	0.05410 (2)	0.00676 (6)	0.68924 (4)	0.01302 (9)	
Cl2	0.05417 (2)	0.44314 (7)	0.18670 (4)	0.01398 (9)	
Cl3	0.05588 (2)	0.22355 (7)	0.42769 (4)	0.01427 (9)	
Cl4	0.18402 (2)	0.04527 (7)	0.03693 (4)	0.01557 (10)	
Cl5	0.25017 (2)	0.38135 (6)	0.17777 (4)	0.01095 (8)	
Cl6	0.32362 (2)	0.06505 (7)	0.07773 (4)	0.01453 (9)	

### Atomic displacement parameters (Å^2^)

**Table:**

	*U*^11^	*U*^22^	*U*^33^	*U*^12^	*U*^13^	*U*^23^
Cs1	0.00923 (5)	0.01059 (5)	0.01374 (6)	0.00077 (4)	0.00290 (4)	0.00089 (4)
Cs2	0.01074 (5)	0.01019 (5)	0.01199 (5)	0.00125 (4)	0.00105 (4)	−0.00113 (4)
Cs3	0.01421 (6)	0.02189 (7)	0.01509 (6)	−0.00445 (5)	0.00429 (5)	−0.00546 (5)
Sc1	0.0070 (2)	0.0084 (2)	0.0079 (2)	0.000	0.00144 (15)	0.000
Sc2	0.0078 (2)	0.0085 (2)	0.0081 (2)	−0.00052 (17)	0.00125 (16)	0.00062 (15)
Cl1	0.0106 (2)	0.0126 (2)	0.0162 (2)	−0.00042 (16)	0.00327 (16)	0.00394 (16)
Cl2	0.0123 (2)	0.0132 (2)	0.0167 (2)	−0.00298 (17)	0.00332 (16)	0.00265 (16)
Cl3	0.0103 (2)	0.0224 (2)	0.00960 (19)	0.00063 (17)	0.00039 (15)	−0.00036 (16)
Cl4	0.0193 (2)	0.0146 (2)	0.0138 (2)	−0.00819 (18)	0.00558 (17)	−0.00093 (16)
Cl5	0.0111 (2)	0.01172 (19)	0.01018 (18)	−0.00123 (16)	0.00216 (14)	−0.00105 (14)
Cl6	0.0148 (2)	0.0147 (2)	0.0134 (2)	0.00510 (18)	0.00040 (16)	0.00119 (16)

### Geometric parameters (Å, º)

**Table:**

Cs1—Cl5^i^	3.3378 (5)	Cs3—Cl6	3.6297 (6)
Cs1—Cl1^ii^	3.3504 (5)	Cs3—Cl1^v^	3.6702 (6)
Cs1—Cl6^iii^	3.3564 (5)	Cs3—Cl3^iii^	3.7752 (6)
Cs1—Cl5	3.3637 (5)	Cs3—Cl2^viii^	3.8144 (6)
Cs1—Cl3	3.4900 (6)	Cs3—Cl1^iii^	3.8312 (6)
Cs1—Cl4^iv^	3.4998 (5)	Cs3—Cl4^vii^	3.8714 (6)
Cs1—Cl2	3.5926 (5)	Cs3—Cl2^i^	3.9852 (6)
Cs1—Cl4	3.6762 (6)	Sc1—Cl3^ix^	2.4718 (5)
Cs2—Cl4^iii^	3.3463 (5)	Sc1—Cl3	2.4718 (5)
Cs2—Cl2^i^	3.3479 (5)	Sc1—Cl2^ix^	2.4940 (6)
Cs2—Cl5	3.4714 (5)	Sc1—Cl2	2.4941 (6)
Cs2—Cl5^i^	3.4937 (5)	Sc1—Cl1^x^	2.5072 (6)
Cs2—Cl3^v^	3.4969 (5)	Sc1—Cl1^ii^	2.5072 (6)
Cs2—Cl6	3.5986 (5)	Sc2—Cl4^vii^	2.4859 (5)
Cs2—Cl6^iv^	3.6000 (6)	Sc2—Cl4	2.4859 (5)
Cs2—Cl1^v^	3.6904 (5)	Sc2—Cl5	2.4889 (5)
Cs3—Cl1^vi^	3.5216 (5)	Sc2—Cl5^vii^	2.4889 (5)
Cs3—Cl2^vii^	3.5559 (6)	Sc2—Cl6	2.4975 (5)
Cs3—Cl3^vi^	3.5878 (6)	Sc2—Cl6^vii^	2.4976 (5)
			
Cl5^i^—Cs1—Cl1^ii^	102.445 (13)	Cl1^x^—Sc1—Cs3^xi^	58.901 (12)
Cl5^i^—Cs1—Cl6^iii^	126.886 (13)	Cl1^ii^—Sc1—Cs3^xi^	126.341 (17)
Cl1^ii^—Cs1—Cl6^iii^	128.714 (13)	Cs1—Sc1—Cs3^xi^	108.888 (3)
Cl5^i^—Cs1—Cl5	81.739 (7)	Cs1^ix^—Sc1—Cs3^xi^	71.484 (3)
Cl1^ii^—Cs1—Cl5	128.226 (13)	Cl3^ix^—Sc1—Cs3^vii^	53.295 (13)
Cl6^iii^—Cs1—Cl5	77.438 (13)	Cl3—Sc1—Cs3^vii^	126.632 (13)
Cl5^i^—Cs1—Cl3	128.060 (13)	Cl2^ix^—Sc1—Cs3^vii^	122.186 (17)
Cl1^ii^—Cs1—Cl3	62.204 (12)	Cl2—Sc1—Cs3^vii^	52.590 (12)
Cl6^iii^—Cs1—Cl3	75.407 (14)	Cl1^x^—Sc1—Cs3^vii^	126.341 (17)
Cl5—Cs1—Cl3	148.367 (13)	Cl1^ii^—Sc1—Cs3^vii^	58.901 (12)
Cl5^i^—Cs1—Cl4^iv^	62.031 (12)	Cs1—Sc1—Cs3^vii^	71.484 (3)
Cl1^ii^—Cs1—Cl4^iv^	96.352 (14)	Cs1^ix^—Sc1—Cs3^vii^	108.888 (3)
Cl6^iii^—Cs1—Cl4^iv^	95.297 (13)	Cs3^xi^—Sc1—Cs3^vii^	173.961 (14)
Cl5—Cs1—Cl4^iv^	128.348 (13)	Cl3^ix^—Sc1—Cs3^xii^	57.081 (16)
Cl3—Cs1—Cl4^iv^	70.483 (13)	Cl3—Sc1—Cs3^xii^	124.00 (2)
Cl5^i^—Cs1—Cl2	159.024 (12)	Cl2^ix^—Sc1—Cs3^xii^	128.462 (13)
Cl1^ii^—Cs1—Cl2	62.017 (12)	Cl2—Sc1—Cs3^xii^	124.235 (13)
Cl6^iii^—Cs1—Cl2	72.479 (13)	Cl1^x^—Sc1—Cs3^xii^	54.672 (15)
Cl5—Cs1—Cl2	96.823 (12)	Cl1^ii^—Sc1—Cs3^xii^	51.268 (14)
Cl3—Cs1—Cl2	59.584 (12)	Cs1—Sc1—Cs3^xii^	103.650 (8)
Cl4^iv^—Cs1—Cl2	130.060 (13)	Cs1^ix^—Sc1—Cs3^xii^	70.532 (5)
Cl5^i^—Cs1—Cl4	68.521 (12)	Cs3^xi^—Sc1—Cs3^xii^	113.557 (8)
Cl1^ii^—Cs1—Cl4	73.931 (13)	Cs3^vii^—Sc1—Cs3^xii^	71.830 (5)
Cl6^iii^—Cs1—Cl4	132.642 (13)	Cl3^ix^—Sc1—Cs3^i^	124.00 (2)
Cl5—Cs1—Cl4	59.605 (12)	Cl3—Sc1—Cs3^i^	57.082 (16)
Cl3—Cs1—Cl4	135.140 (12)	Cl2^ix^—Sc1—Cs3^i^	124.235 (13)
Cl4^iv^—Cs1—Cl4	126.021 (7)	Cl2—Sc1—Cs3^i^	128.461 (13)
Cl2—Cs1—Cl4	92.585 (13)	Cl1^x^—Sc1—Cs3^i^	51.268 (14)
Cl4^iii^—Cs2—Cl2^i^	144.147 (14)	Cl1^ii^—Sc1—Cs3^i^	54.672 (15)
Cl4^iii^—Cs2—Cl5	70.962 (12)	Cs1—Sc1—Cs3^i^	70.532 (5)
Cl2^i^—Cs2—Cl5	131.312 (13)	Cs1^ix^—Sc1—Cs3^i^	103.650 (8)
Cl4^iii^—Cs2—Cl5^i^	114.518 (14)	Cs3^xi^—Sc1—Cs3^i^	71.830 (5)
Cl2^i^—Cs2—Cl5^i^	99.045 (13)	Cs3^vii^—Sc1—Cs3^i^	113.557 (8)
Cl5—Cs2—Cl5^i^	78.039 (6)	Cs3^xii^—Sc1—Cs3^i^	67.059 (8)
Cl4^iii^—Cs2—Cl3^v^	72.190 (13)	Cl4^vii^—Sc2—Cl4	179.999 (18)
Cl2^i^—Cs2—Cl3^v^	76.407 (13)	Cl4^vii^—Sc2—Cl5	90.268 (17)
Cl5—Cs2—Cl3^v^	140.539 (13)	Cl4—Sc2—Cl5	89.731 (17)
Cl5^i^—Cs2—Cl3^v^	131.180 (12)	Cl4^vii^—Sc2—Cl5^vii^	89.730 (17)
Cl4^iii^—Cs2—Cl6	128.042 (13)	Cl4—Sc2—Cl5^vii^	90.271 (17)
Cl2^i^—Cs2—Cl6	72.498 (12)	Cl5—Sc2—Cl5^vii^	180.0
Cl5—Cs2—Cl6	60.126 (12)	Cl4^vii^—Sc2—Cl6	86.646 (19)
Cl5^i^—Cs2—Cl6	72.674 (12)	Cl4—Sc2—Cl6	93.354 (19)
Cl3^v^—Cs2—Cl6	143.761 (13)	Cl5—Sc2—Cl6	90.573 (16)
Cl4^iii^—Cs2—Cl6^iv^	93.629 (13)	Cl5^vii^—Sc2—Cl6	89.427 (16)
Cl2^i^—Cs2—Cl6^iv^	92.949 (14)	Cl4^vii^—Sc2—Cl6^vii^	93.356 (19)
Cl5—Cs2—Cl6^iv^	123.475 (13)	Cl4—Sc2—Cl6^vii^	86.644 (19)
Cl5^i^—Cs2—Cl6^iv^	59.259 (11)	Cl5—Sc2—Cl6^vii^	89.427 (16)
Cl3^v^—Cs2—Cl6^iv^	72.323 (12)	Cl5^vii^—Sc2—Cl6^vii^	90.574 (16)
Cl6—Cs2—Cl6^iv^	126.850 (7)	Cl6—Sc2—Cl6^vii^	180.0
Cl4^iii^—Cs2—Cl1^v^	73.789 (14)	Cl4^vii^—Sc2—Cs1^ii^	132.946 (12)
Cl2^i^—Cs2—Cl1^v^	76.813 (13)	Cl4—Sc2—Cs1^ii^	47.055 (12)
Cl5—Cs2—Cl1^v^	93.390 (12)	Cl5—Sc2—Cs1^ii^	136.773 (12)
Cl5^i^—Cs2—Cl1^v^	164.523 (12)	Cl5^vii^—Sc2—Cs1^ii^	43.228 (12)
Cl3^v^—Cs2—Cl1^v^	62.887 (12)	Cl6—Sc2—Cs1^ii^	92.711 (13)
Cl6—Cs2—Cl1^v^	91.899 (13)	Cl6^vii^—Sc2—Cs1^ii^	87.288 (13)
Cl6^iv^—Cs2—Cl1^v^	135.208 (12)	Cl4^vii^—Sc2—Cs1^iii^	47.054 (12)
Cl1^vi^—Cs3—Cl2^vii^	121.939 (13)	Cl4—Sc2—Cs1^iii^	132.945 (12)
Cl1^vi^—Cs3—Cl3^vi^	63.717 (12)	Cl5—Sc2—Cs1^iii^	43.227 (12)
Cl2^vii^—Cs3—Cl3^vi^	58.283 (12)	Cl5^vii^—Sc2—Cs1^iii^	136.772 (12)
Cl1^vi^—Cs3—Cl6	148.943 (13)	Cl6—Sc2—Cs1^iii^	87.289 (13)
Cl2^vii^—Cs3—Cl6	89.091 (12)	Cl6^vii^—Sc2—Cs1^iii^	92.712 (13)
Cl3^vi^—Cs3—Cl6	147.118 (12)	Cs1^ii^—Sc2—Cs1^iii^	180.0
Cl1^vi^—Cs3—Cl1^v^	57.631 (15)	Sc1^x^—Cl1—Cs1^iv^	91.260 (16)
Cl2^vii^—Cs3—Cl1^v^	173.337 (13)	Sc1^x^—Cl1—Cs3^xi^	94.994 (16)
Cl3^vi^—Cs3—Cl1^v^	121.151 (12)	Cs1^iv^—Cl1—Cs3^xi^	173.698 (17)
Cl6—Cs3—Cl1^v^	91.731 (12)	Sc1^x^—Cl1—Cs3^v^	91.457 (16)
Cl1^vi^—Cs3—Cl3^iii^	57.741 (11)	Cs1^iv^—Cl1—Cs3^v^	91.711 (12)
Cl2^vii^—Cs3—Cl3^iii^	116.971 (12)	Cs3^xi^—Cl1—Cs3^v^	87.364 (13)
Cl3^vi^—Cs3—Cl3^iii^	88.872 (13)	Sc1^x^—Cl1—Cs2^v^	168.727 (19)
Cl6—Cs3—Cl3^iii^	111.892 (12)	Cs1^iv^—Cl1—Cs2^v^	78.623 (11)
Cl1^v^—Cs3—Cl3^iii^	56.679 (11)	Cs3^xi^—Cl1—Cs2^v^	95.079 (12)
Cl1^vi^—Cs3—Cl2^viii^	73.210 (12)	Cs3^v^—Cl1—Cs2^v^	83.964 (11)
Cl2^vii^—Cs3—Cl2^viii^	89.108 (12)	Sc1^x^—Cl1—Cs3^i^	87.017 (15)
Cl3^vi^—Cs3—Cl2^viii^	69.717 (12)	Cs1^iv^—Cl1—Cs3^i^	89.870 (13)
Cl6—Cs3—Cl2^viii^	109.110 (12)	Cs3^xi^—Cl1—Cs3^i^	91.227 (12)
Cl1^v^—Cs3—Cl2^viii^	96.862 (12)	Cs3^v^—Cl1—Cs3^i^	177.828 (16)
Cl3^iii^—Cs3—Cl2^viii^	130.920 (11)	Cs2^v^—Cl1—Cs3^i^	97.813 (13)
Cl1^vi^—Cs3—Cl1^iii^	88.772 (12)	Sc1—Cl2—Cs2^iii^	164.21 (2)
Cl2^vii^—Cs3—Cl1^iii^	57.881 (12)	Sc1—Cl2—Cs3^vii^	93.554 (16)
Cl3^vi^—Cs3—Cl1^iii^	56.711 (11)	Cs2^iii^—Cl2—Cs3^vii^	89.963 (13)
Cl6—Cs3—Cl1^iii^	111.495 (11)	Sc1—Cl2—Cs1	86.012 (16)
Cl1^v^—Cs3—Cl1^iii^	115.801 (4)	Cs2^iii^—Cl2—Cs1	78.554 (11)
Cl3^iii^—Cs3—Cl1^iii^	59.122 (11)	Cs3^vii^—Cl2—Cs1	90.658 (13)
Cl2^viii^—Cs3—Cl1^iii^	125.895 (12)	Sc1—Cl2—Cs3^xiii^	99.566 (17)
Cl1^vi^—Cs3—Cl4^vii^	120.934 (12)	Cs2^iii^—Cl2—Cs3^xiii^	95.760 (13)
Cl2^vii^—Cs3—Cl4^vii^	89.959 (12)	Cs3^vii^—Cl2—Cs3^xiii^	90.890 (12)
Cl3^vi^—Cs3—Cl4^vii^	123.282 (12)	Cs1—Cl2—Cs3^xiii^	174.107 (16)
Cl6—Cs3—Cl4^vii^	54.119 (11)	Sc1—Cl2—Cs3^iii^	95.280 (17)
Cl1^v^—Cs3—Cl4^vii^	85.176 (11)	Cs2^iii^—Cl2—Cs3^iii^	83.870 (12)
Cl3^iii^—Cs3—Cl4^vii^	63.654 (11)	Cs3^vii^—Cl2—Cs3^iii^	167.614 (16)
Cl2^viii^—Cs3—Cl4^vii^	163.219 (12)	Cs1—Cl2—Cs3^iii^	98.594 (12)
Cl1^iii^—Cs3—Cl4^vii^	66.677 (11)	Cs3^xiii^—Cl2—Cs3^iii^	79.100 (11)
Cl1^vi^—Cs3—Cl2^i^	96.304 (12)	Sc1—Cl3—Cs1	88.641 (15)
Cl2^vii^—Cs3—Cl2^i^	116.554 (5)	Sc1—Cl3—Cs2^v^	163.83 (2)
Cl3^vi^—Cs3—Cl2^i^	123.146 (12)	Cs1—Cl3—Cs2^v^	81.079 (11)
Cl6—Cs3—Cl2^i^	65.179 (11)	Sc1—Cl3—Cs3^xi^	93.175 (15)
Cl1^v^—Cs3—Cl2^i^	69.674 (11)	Cs1—Cl3—Cs3^xi^	177.893 (16)
Cl3^iii^—Cs3—Cl2^i^	126.317 (11)	Cs2^v^—Cl3—Cs3^xi^	97.376 (13)
Cl2^viii^—Cs3—Cl2^i^	53.436 (15)	Sc1—Cl3—Cs3^i^	89.577 (19)
Cl1^iii^—Cs3—Cl2^i^	174.105 (11)	Cs1—Cl3—Cs3^i^	87.819 (13)
Cl4^vii^—Cs3—Cl2^i^	112.763 (11)	Cs2^v^—Cl3—Cs3^i^	102.381 (13)
Cl3^ix^—Sc1—Cl3	178.91 (4)	Cs3^xi^—Cl3—Cs3^i^	91.125 (13)
Cl3^ix^—Sc1—Cl2^ix^	90.284 (19)	Sc2—Cl4—Cs2^i^	150.42 (2)
Cl3—Sc1—Cl2^ix^	88.945 (19)	Sc2—Cl4—Cs1^ii^	101.617 (17)
Cl3^ix^—Sc1—Cl2	88.945 (19)	Cs2^i^—Cl4—Cs1^ii^	83.078 (12)
Cl3—Sc1—Cl2	90.28 (2)	Sc2—Cl4—Cs1	100.906 (16)
Cl2^ix^—Sc1—Cl2	89.45 (3)	Cs2^i^—Cl4—Cs1	78.879 (11)
Cl3^ix^—Sc1—Cl1^x^	90.480 (19)	Cs1^ii^—Cl4—Cs1	157.264 (17)
Cl3—Sc1—Cl1^x^	90.306 (19)	Sc2—Cl4—Cs3^vii^	106.150 (17)
Cl2^ix^—Sc1—Cl1^x^	91.511 (18)	Cs2^i^—Cl4—Cs3^vii^	103.290 (14)
Cl2—Sc1—Cl1^x^	178.89 (2)	Cs1^ii^—Cl4—Cs3^vii^	86.175 (12)
Cl3^ix^—Sc1—Cl1^ii^	90.305 (19)	Cs1—Cl4—Cs3^vii^	84.641 (13)
Cl3—Sc1—Cl1^ii^	90.479 (19)	Sc2—Cl5—Cs1^iii^	106.063 (17)
Cl2^ix^—Sc1—Cl1^ii^	178.89 (2)	Sc2—Cl5—Cs1	109.697 (16)
Cl2—Sc1—Cl1^ii^	91.510 (18)	Cs1^iii^—Cl5—Cs1	144.225 (16)
Cl1^x^—Sc1—Cl1^ii^	87.54 (3)	Sc2—Cl5—Cs2	106.525 (16)
Cl3^ix^—Sc1—Cs1	124.476 (13)	Cs1^iii^—Cl5—Cs2	82.000 (11)
Cl3—Sc1—Cs1	55.599 (13)	Cs1—Cl5—Cs2	88.842 (12)
Cl2^ix^—Sc1—Cs1	127.855 (17)	Sc2—Cl5—Cs2^iii^	107.265 (15)
Cl2—Sc1—Cs1	57.945 (12)	Cs1^iii^—Cl5—Cs2^iii^	88.886 (12)
Cl1^x^—Sc1—Cs1	121.740 (17)	Cs1—Cl5—Cs2^iii^	79.759 (11)
Cl1^ii^—Sc1—Cs1	52.385 (11)	Cs2—Cl5—Cs2^iii^	146.210 (15)
Cl3^ix^—Sc1—Cs1^ix^	55.600 (13)	Sc2—Cl6—Cs1^i^	136.14 (2)
Cl3—Sc1—Cs1^ix^	124.478 (13)	Sc2—Cl6—Cs2	102.768 (17)
Cl2^ix^—Sc1—Cs1^ix^	57.945 (12)	Cs1^i^—Cl6—Cs2	78.359 (11)
Cl2—Sc1—Cs1^ix^	127.855 (17)	Sc2—Cl6—Cs2^ii^	104.044 (16)
Cl1^x^—Sc1—Cs1^ix^	52.385 (11)	Cs1^i^—Cl6—Cs2^ii^	81.426 (12)
Cl1^ii^—Sc1—Cs1^ix^	121.740 (17)	Cs2—Cl6—Cs2^ii^	153.152 (16)
Cs1—Sc1—Cs1^ix^	173.309 (14)	Sc2—Cl6—Cs3	113.085 (18)
Cl3^ix^—Sc1—Cs3^xi^	126.634 (13)	Cs1^i^—Cl6—Cs3	110.735 (14)
Cl3—Sc1—Cs3^xi^	53.296 (13)	Cs2—Cl6—Cs3	85.863 (12)
Cl2^ix^—Sc1—Cs3^xi^	52.590 (13)	Cs2^ii^—Cl6—Cs3	84.957 (13)
Cl2—Sc1—Cs3^xi^	122.185 (17)		

Symmetry codes: (i) −*x*+1/2, *y*−1/2, −*z*+1/2; (ii) *x*, −*y*, *z*−1/2; (iii) −*x*+1/2, *y*+1/2, −*z*+1/2; (iv) *x*, −*y*, *z*+1/2; (v) −*x*+1/2, −*y*+1/2, −*z*+1; (vi) *x*+1/2, −*y*+1/2, *z*−1/2; (vii) −*x*+1/2, −*y*+1/2, −*z*; (viii) *x*+1/2, *y*−1/2, *z*; (ix) −*x*, *y*, −*z*+1/2; (x) −*x*, −*y*, −*z*+1; (xi) *x*−1/2, −*y*+1/2, *z*+1/2; (xii) *x*−1/2, *y*−1/2, *z*; (xiii) *x*−1/2, *y*+1/2, *z*.
